# Clinical characteristics of children with MIS-C fulfilling classification criteria for macrophage activation syndrome

**DOI:** 10.3389/fped.2022.981711

**Published:** 2022-09-15

**Authors:** Piotr Buda, Ewa Strauss, Danuta Januszkiewicz-Lewandowska, Ewa Czerwinska, Kamila Ludwikowska, Leszek Szenborn, Ewelina Gowin, Magdalena Okarska-Napierała, Ernest Kuchar, Janusz Ksia̧zyk

**Affiliations:** ^1^Department of Pediatrics, Nutrition and Metabolic Diseases, Children's Memorial Health Institute, Warsaw, Poland; ^2^Institute of Human Genetics, Polish Academy of Sciences, Poznan, Poland; ^3^Department of Pediatric Oncology, Hematology and Transplantology, Poznan University of Medical Sciences, Poznan, Poland; ^4^Department of Pediatric Infectious Diseases, Wroclaw Medical University, Wroclaw, Poland; ^5^Health Promotion Department, Poznan University of Medical Sciences, Poznan, Poland; ^6^Department of Pediatrics With Clinical Decisions Unit, Medical University of Warsaw, Warsaw, Poland

**Keywords:** macrophage activation syndrome, MAS, SARS-CoV-2, pediatric inflammatory multisystem syndrome temporally associated with SARS-CoV-2 (PIMS-TS), PIMS-TS, multisystem inflammatory syndrome in children (MIS-C), MIS-C, Kawasaki disease (KD)

## Abstract

**Background:**

Macrophage activation syndrome (MAS) is a potentially life-threatening complication of various inflammatory disorders, including multisystem inflammatory syndrome in children (MIS-C). MIS-C refractory to treatment should raise suspicion of MAS, which can be fatal if a definitive diagnosis is delayed. Unfortunately, there is a lack of data on MAS in children with MIS-C.

**Objective:**

Our study aims to analyze the risk factors for the development of MAS in MIS-C, its clinical course and response to treatment, and identify predictive factors for pediatric intensive care.

**Material and methods:**

We analyzed data from the Polish MIS-C registry of the MultiOrgan Inflammatory Syndromes COVID-19 Related Study. Patients were diagnosed according to the WHO MIS-C definition and treated according to national guidelines (Polish Pediatric Society) based on international consensus. MAS definition was based on 2016 Classification Criteria for Macrophage Activation Syndrome Complicating Systemic Juvenile Idiopathic Arthritis.

**Results:**

Two-hundred and seventy four children met the study inclusion criteria. Fifty-nine patients fulfilled MAS classification criteria, nine of which required admission to the pediatric intensive care unit (PICU). MIS-C patients with MAS were significantly older than patients without MAS (median 11.2 vs. 8.1 years). Multivariable analysis showed that age, symptoms characteristic of atypical Kawasaki disease, and skin erosions were significant factors associated with MAS in MIS-C patients. Analysis of laboratory parameters showed that on admission, MIS-C patients with MAS had significantly lower median lymphocyte and platelet counts, albumin and sodium levels, and higher median levels of C-reactive protein, procalcitonin, ferritin, D-dimers, triglycerides, serum creatinine, urea, and γ-glutamyl transpeptidase, and neutrophil count. Multivariate analysis showed that higher procalcitonin, ferritin, and fibrinogen levels at admission were predictive of MAS. Only elevated troponin level was a factor indicating a requirement of PICU hospitalization for children with MAS. MIS-C patients fulfilling MAS criteria were treated more often with intravenous immunoglobulins and steroids than children without MAS. Children with MAS more often required mechanical ventilation. None of the patients required biological agents.

**Conclusions:**

The clinical course of MAS in MIS-C seems milder, treatment less aggressive, and the prognosis better than expected based on the current knowledge on MAS complicating other rheumatological diseases.

## Introduction

Multisystem inflammatory syndrome in children (MIS-C) or pediatric inflammatory multisystem syndrome temporally associated with SARS-CoV-2 (PIMS-TS) is a new condition (reported in April 2020) associated with the ongoing coronavirus disease 2019 (COVID-19) pandemic, (1–5).

MIS-C develops approximately 4 weeks after symptomatic or asymptomatic SARS-CoV-2 infection (6, 7). It involves hyperinflammatory multisystem damage due to immune dysregulation. Although, the exact pathomechanism of this late COVID-19 complication remains unknown. Several hypotheses include: superantigenic stimulation (8, 9); lymphocyte exhaustion due to chronic SARS-CoV-2 exposure (10–12); increased intestine permeability and translocation of the virus particles into the circulation (13); and autoantibodies production (10, 14–17). Genetic susceptibility probably plays a role, too (18, 19). Immune dysregulation of MIS-C involves activation of neutrophils, macrophages, and dendritic cells with robust chemokine and cytokine production (10, 14, 20), profound lymphopenia (10), decreased IFN-α and increased IFN-γ (IFN-γ) responses (9, 10, 20).

MIS-C is characterized by fever, multisystem organ involvement (gastrointestinal, cardiovascular, neurologic, respiratory, mucocutaneous, renal, and hematologic symptoms), and laboratory findings indicating severe inflammation. Precise diagnostic and treatment guidelines were published by different health organizations (21–24). Polish recommendations were proposed based on international publications and adjusted to the local data (25). MIS-C can have various presentations - from mild, self-limiting disease to severe, life-threatening illness requiring pediatric intensive care unit (PICU) hospitalization. The complications usually involve the cardiovascular system (2, 3, 26). The treatment is based on immunomodulating agents – intravenous immunoglobulins (IVIG), steroids or biological therapy, and supportive care (21, 22, 25).

Although MIS-C has specific epidemiological and clinical features, it may resemble other inflammatory disorders like Kawasaki disease (KD), sepsis, and toxic shock syndrome. Since the beginning, the similarity to macrophage activation syndrome (MAS) has also been observed (2, 3, 17).

MAS is a secondary hemophagocytic lymphohistiocytosis (HLH) associated with autoimmune diseases (27). Usually, MAS is described as a severe exacerbation or one of the most serious complications of connective tissue diseases, particularly systemic juvenile idiopathic arthritis (s-JIA). MAS has also been associated with KD, but the exact incidence is unknown (28, 29). There is a lack of data on MAS in children with COVID-19 and MIS-C. In one of the first MIS-C reports, the incidence is as high as 50% of MIS-C children (2).

MAS is a life-threatening systemic extreme-inflammatory syndrome caused by multifactorial immune dysregulation and pathological hyperactivation of the immune system. The main symptoms include fever and hepatosplenomegaly. They can be followed by coagulopathy and circulatory, respiratory, and multiorgan failure, with mortality rates as high as 8–22 % in children with s-JIA (30, 31). It is challenging to distinguish MAS from other complications (such as s-JIA flares, sepsis-like syndromes, and autoinflammatory disorders) because there are no specific clinical or laboratory markers. KD, s-JIA, and MAS share many common clinical and immunologic features (32).

The MAS criteria are validated for s-JIA (2016 Classification Criteria for Macrophage Activation Syndrome Complicating Systemic Juvenile Idiopathic Arthritis) but are commonly used for other systemic autoinflammatory diseases such as KD and other pediatric rheumatic diseases pediatric (33, 34). Some pediatricians use the criteria of a histiocytic society instead (HLH-2004) (35–37). However, according to Davi et al., they are likely not appropriate for identifying MAS in children with s-JIA because they are intended primarily for genetic forms of HLH (38). MAS may frequently be under-recognized in children with KD because there are no distinct criteria for MAS complicating KD (39). Establishing proper criteria for MAS in different entities is crucial, as it is a severe, life-threatening condition requiring prompt and aggressive immunomodulatory treatment (27, 35). The main goal is to implement MAS treatment as soon as possible to stop the “cytokine storm.” The therapy depends on the underlying disease and the experience of clinical centers (35, 37).

Because there are no specific criteria for diagnosis of MAS in MIS-C and given clinical and laboratory features of MIS-C that resemble MAS, 2016 Classification Criteria for Macrophage Activation Syndrome Complicating Systemic Juvenile Idiopathic Arthritis were previously applied to MIS-C (2, 40, 41).

Our study aims to (i) identify risk factors for developing MAS (according to 2016 MAS criteria) in the course of MIS-C; (ii) characterize its clinical presentation and response to treatment; and (iii) identify predictive factors for the need for intensive care in this subgroup of patients.

## Materials and methods

### Patients

We analyzed the data from the Polish MIS-C registry of the MultiOrgan Inflammatory Syndromes COVID-19 Related (MOIS-CoR) Study, including the period from March 4, 2020 (when the first COVID-19 case in Poland was confirmed) to April 20, 2021 (42). Anonymous patient data from 42 pediatric hospitals across the country were extracted from patient records and collected using a web-based form developed for this purpose. Registry enrolment of patients did not influence decisions for treatment or PICU admission. Ethical approval was obtained from the Bioethics Committee at the Wroclaw Medical University (CWN UMW BW: 313/2020).

### Inclusion criteria

The inclusion criteria for the MOIS-CoR registry and patient eligibility have been presented previously (42). We adopted the WHO definition of MIS-C. MAS was diagnosed based on the 2016 Classification Criteria for Macrophage Activation Syndrome Complicating Systemic Juvenile Idiopathic Arthritis (33). MIS-C and MAS definitions were established based on the respective peak values of laboratory findings during the hospitalization. We also verified the diagnosis of MAS based on clinical HLH-2004 criteria (except for the concentration of soluble CD25 and natural killer cell activity because these tests were unavailable) (36).

**Figure 1:**
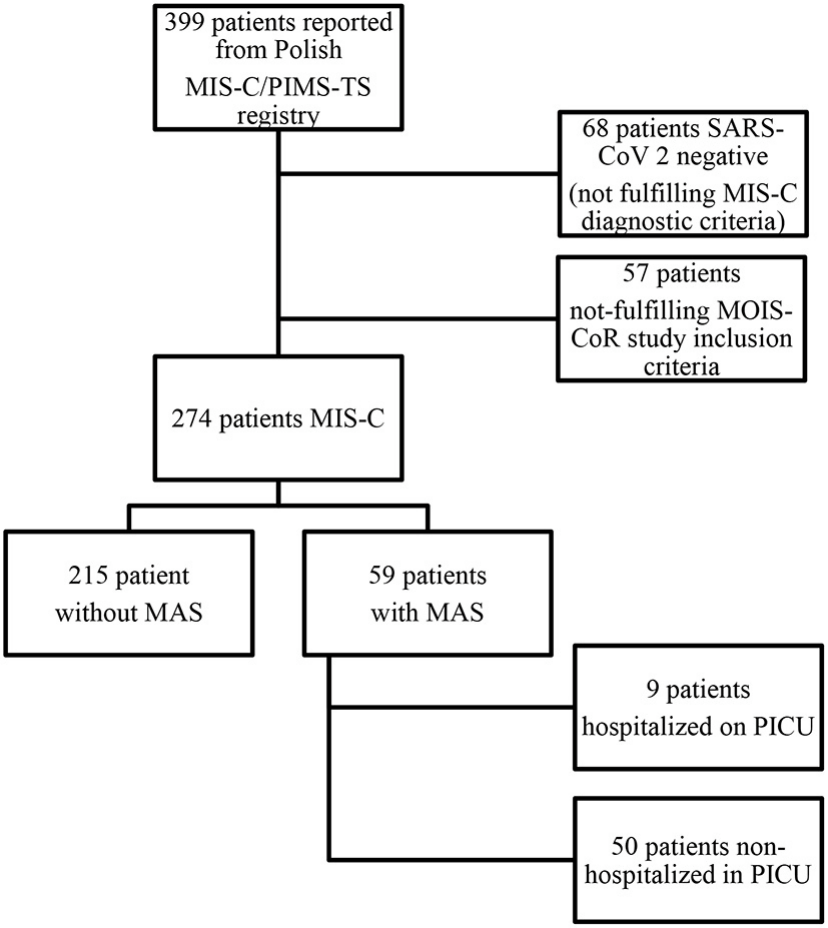
Study flow diagram illustrating the patients' inclusion process for the study.

The selected group of 274 patients was divided into two subgroups - one fulfilling the criteria for MAS and the other without MAS. Children with MAS were additionally divided according to the need for PICU hospitalization. Figure 1 shows the patient inclusion process for the study.

### Demographic, clinical, and laboratory data

Demographic characteristics, clinical symptoms, treatment, and outcomes were compared in all subgroups. In addition, laboratory results on admission and at peak (minimum or maximum) were also analyzed. The study's standard definitions and measures, including laboratory and echocardiographic abnormalities, level of consciousness, and nutritional status, have been described in detail in a previous study (42).

### Statistical analysis

Univariate analyses used the *t*-test or Fisher exact test for quantitative parameters and the χ2 test for qualitative parameters. Continuous parameters with non-normal distribution were analyzed using the Mann-Whitney U test. Normality of distribution was assessed using the Shapiro Wilk test. Logistic regression analysis was used for multivariate analysis, including adjustment for age. Factors deviating from the normal distribution after logarithmic transformation or after conversion to quartiles were examined through these analyses. Analyses were performed using Statistica v10.0 software. Observed differences were considered significant at *P* < 0.05.

## Results

### Entire study group

Out of 399 children enrolled in the Polish registry with suspected MIS-C, 274 met the WHO criteria of MIS-C. Fifty-nine patients fulfilled MAS 2016 classification criteria, including nine patients who required admission to PICU (Figure 1). None of the patients with MAS or MIS-C in the whole group met the criteria for hemophagocytic lymphohistiocytosis, according to HLH-2004.

### Comparison of groups of children with and without MAS criteria fulfillment

#### Clinical characteristics

MIS-C patients fulfilling MAS criteria were significantly older than patients without MAS (median 11.2 vs. 8.1 years). For this reason, the age-adjusted analysis was done. MAS was most common in children aged 5–12 years (50.9%) and in MIS-C children with the KD phenotype (57.7%), especially for its atypical form (76.3%). In addition, MIS-C patients with MAS were more likely to have an AVPU score (alert/verbal/pain/unresponsive consciousness assessment scale) below A (12.7 vs. 4.8%), swelling or erythema of the hands and feet (75.5 vs. 49.8%), respiratory distress (41.8 vs. 19.4%), joint pain (32.7 vs. 15.7%), dysuria (27.8 vs. 12.3%), and skin erosions (including chilblains-like phenomenon affecting fingers and toes) (9.6 vs. 1.5%). A small percentage of children had comorbidities, with no significant difference in patients with MAS. Detailed clinical data in the analyzed patient subgroups, including those requiring PICU hospitalization, are presented in Supplementary Table 1. Multivariable analysis showed that greater age, symptoms characteristic of atypical KD, and skin erosion were significantly more common among children with MAS in MIS-C (Table 1).

**Table 1:** Multivariate analysis of the association between symptom occurrence and the presence of macrophage activation syndrome (MAS) or atypical Kawasaki disease (KD) in 274 patients meeting MIS-C criteria.

**Parameter** **[analyzed value]**	**MAS**	**Atypical KD**
Age [1 y]	1.2 (1.1; 1.4); *P* <0.001	NS
Atypical KD	4.6 (2.2–9.5); *P* < 0.0001	NA
MAS	NA	2.6 (1.3–5.2); *P* < 0.009
Hands and feet swelling or erythema	NA	4.2 (2.4–3.4); *P <* 0.0001
Skin peeling on digits	NA	2,6 (1.1–6.2); *P =* 0,024
Skin erosions	6.3 (1.1– 35,5); *P =* 0.036	NA
Model summary (accuracy)	OR = 7.3; 80.3%; *P* < 0.0001 (96.3% non–MAS; 22.0% MAS)	OR = 4.9; 68.6%; *P* < 0.0001 (56.5% non–Atypical KD; 78.7% Atypical KD)

#### Laboratory results at admission

Analysis of laboratory parameters showed that on admission, MIS-C patients fulfilling MAS criteria had already significantly lower median of lymphocyte (0.74 vs. 1.16 10^3^/μL) and platelet (140 vs. 188 10^3^/μL) counts, albumin (3.1 vs. 3.4 g/dL), and sodium (132 vs. 135 mmol/L) levels compared with patients without MAS. In addition, children with MAS had significantly higher median concentrations of C-reactive protein (CRP) (189.08 vs. 129 mg/L), procalcitonin (8.35 vs. 1.855 ng/mL), ferritin (920.13 vs. 292.3 μg/L), D-dimers (3.78 vs. 2.4 mg/L), triglycerides (210 vs. 140 mg/dL), serum creatinine (0.65 vs. 0.48 mg/dL), urea (27 vs. 22 mg/dL), and activity of γ-glutamyl transpeptidase (40 vs. 20 U/L), and neutrophil count (9.4 vs. 7.04 10^3^/μL). There were also significantly higher median plasma concentrations of bilirubin, B-type natriuretic peptide (BNP) and N-terminal pro-B-type natriuretic peptide (NT-proBNP), troponin, and alanine and aspartate transaminases activities, and estimated glomerular filtration rate (eGFR) in MIS-C patients with MAS. However, the age-adjusted analysis did not confirm any of these differences. Detailed data of laboratory results in MIS-C patients with and without MAS on admission are presented in Supplementary Table 1.

Multivariate analysis of the association between laboratory findings on the admission and MAS criteria fulfillment among 274 children with MIS-C showed that older age, higher procalcitonin, ferritin, and fibrinogen levels were indicative of MAS.

#### Laboratory results at respective peaks

Similar results were obtained by analyzing laboratory parameters at specific peak points in a group of MIS-C children with or without MAS criteria fulfillment. Detailed data are summarized in Supplementary Table 3.

Multivariate analysis showed that elevated leukocyte counts and ferritin plasma concentration were associated with MAS (Table 3).

### Comparison of groups of children with MAS criteria fulfillment that required and not required admission to PICU

#### Clinical characteristics

Among the demographic and clinical characteristics of admission of MIS-C patients with MAS, only hypotension on admission was significantly more common in patients who required treatment in the PICU than other MIS-C children with MAS (62.5 vs. 15.8%) (Supplementary Table 1).

#### Laboratory results at admission

Compared to patients who did not require PICU hospitalization, MIS-C children who finally fulfilled MAS criteria and were treated in the PICU had exceptional laboratory results on admission: significantly higher median leukocyte (13.51 vs. 10.24 10^3^/μL), neutrophil counts (12.4 vs. 8.56 10^3^/μL), and concentrations of procalcitonin (49.87 vs. 6.1 ng/mL), NT-proBNP (32,672 vs. 3,174 pg/mL), serum creatinine (1.4 vs. 0.57 mg/dL), urea (61 vs. 25.5 mg/dL), and fibrinogen (7.14 vs. 5.51 g/L). All children with MAS requiring PICU had elevated serum troponin levels.

**Figure 2:**
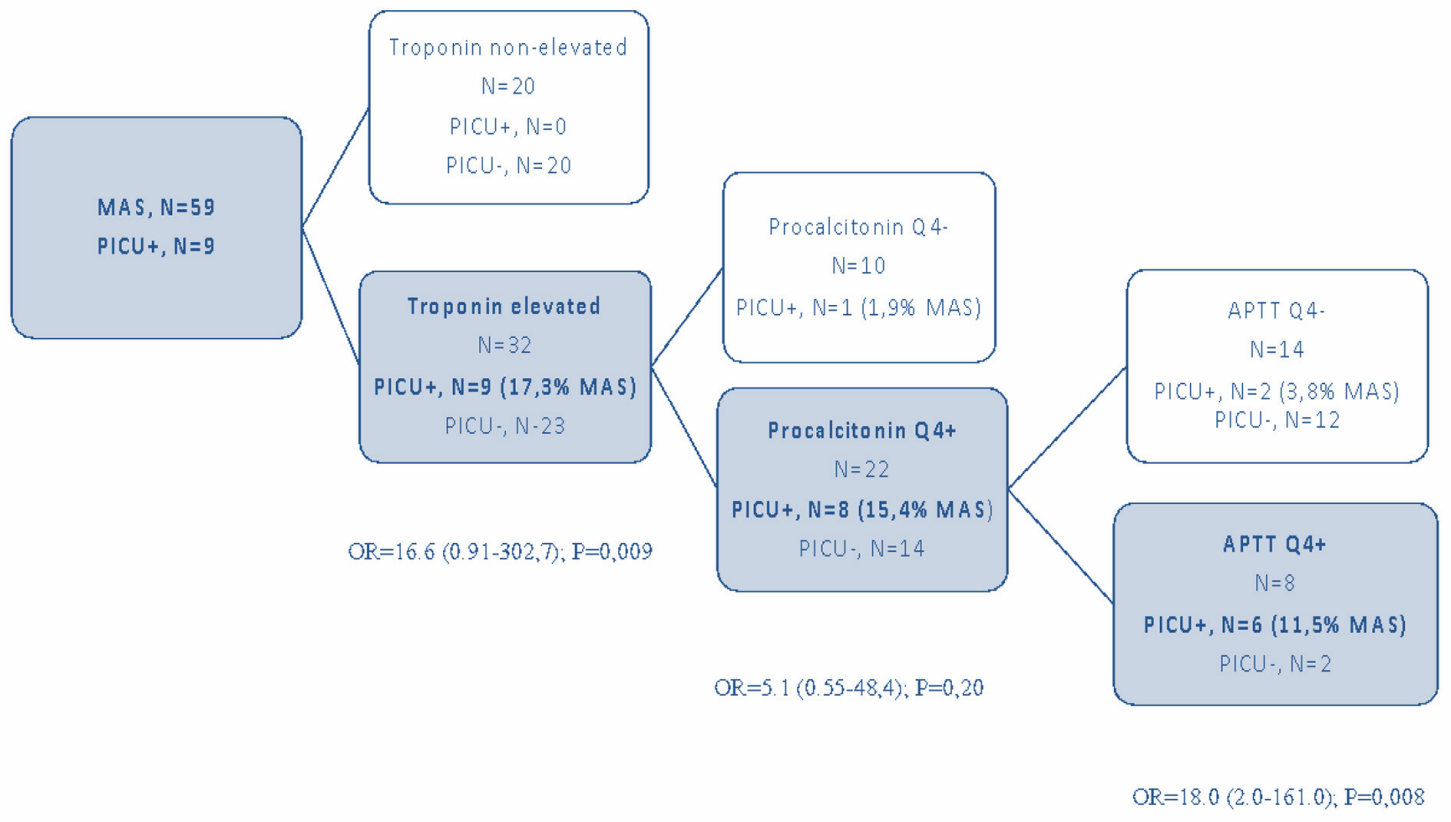
Predictive factors for PICU hospitalization in children with MIS-C and MAS.

Multivariate analysis showed that only elevated troponin level was a predictive factor (Table 2, Figure 2).

**Table 2:** Multivariate analysis of the association between laboratory findings on hospital admission and the presence of macrophage activation syndrome (MAS) and MAS requiring pediatric intensive care unit (PICU) hospitalization (MAS–PICU) in 274 patients meeting MIS–C criteria.

**Parameter** **[analyzed value]**	**MAS *N* = 274**	**MAS–PICU** ***N*** = **59**	
		**Model 1**	**Model 2**
Age [1 y]	1.2 (1.1–1.3); *P =* 0.001	NA	NA
Procalcitonin (ng/mL) [Q 1–4]	1.6 (1.1–2.2); *P =* 0.018	4.8 (0.93–25.0); *P =* 0.055	NA
Ferritin (μg/L) [Q 1–4]	6.2 (3.6–10.6); *P* < 0.00001	NA	NA
Fibrinogen (g/L) [Q 1–4]	NA	2.4 (1.0–5.8); *P =* 0.046	NA
Elevated level of troponin (ng/L)	NA	NA	31.2 (1.7–578.7); *P =* 0.0004
Model summary (accuracy)	OR = 33.0; Accuracy 88.3% *P* < 0.0001 Controls 94.4% cases 66.1%	NE; *P =* 0.004	NE; *P =* 0.004

NA, not analyzed; NE, not estimable.

#### Laboratory results at a respective peak

Similar results were obtained by analyzing laboratory parameters in the subsequent course of the disease (at its peak). Patients not hospitalized in PICU had a shorter APTT than children admitted to PICU. A detailed analysis of the results is presented in Supplementary Tables 4, 5.

Multivariate analysis showed that high troponin, procalcitonin, and APTT prolongation were significantly more frequently observed in patients with MAS requiring treatment in the PICU (Table 3).

**Table 3:** Multivariate analysis of the association between laboratory results at respective peaks and the presence of macrophage activation syndrome (MAS) and MAS requiring pediatric intensive care unit (PICU) hospitalization (MAS-PICU) in 274 patients meeting MIS-C criteria.

**Parameter** **[analyzed value]**	**MAS *N* = 274**	**MAS –PICU** ***N*** **= 59**	
		**Model 1**	**Model 2**
Ferritin max (μg/L) [Q 1–4]	52.6 (19.3–143.3); *P* < 0.00001	NA	NA
WBC (10^3^/uL) [Q 1–4]	1.4 (0.89–2.4); *P =* 0.136	NA	NA
Procalcitonin (ng/mL) [Q 1–4]	NA	12.1 (1.2–123.9); *P =* 0.032	NA
APTT (s) [Q 1–4]	NA	3.3 (1.2–9.1); *P =* 0.019	NA
Elevated level of troponin at its max (ng/L)	NA	NA	31.2 (1.7–578.7); *P =* 0.0004
Model summary (accuracy)	OR = 577.5; 96.7%; *P* < 0.0001 (non-MAS 97.7%. MAS 93.2%)	OR=23.0; 88.1%; *P =* 0.003 (MAS-non PICU 92.0%. MAS-PICU 66.7%	NE; *P =* 0.004

NA, not analyzed; NE, not estimable.

### Therapy and outcome

Various treatment options were used and adjusted to current guidelines (Supplementary Table 6). MIS-C patients fulfilling MAS criteria were treated more often with intravenous immunoglobulins (94.8 vs. 87.6%) and steroids (87.3 vs. 62.7%) than children without MAS. Children with MAS more often required mechanical ventilation (10.9 vs. 1.9%). No significant differences in such treatment when comparing groups of children with MAS only vs. those hospitalized in PICU were found. None of the patients required biologic agents. Therapeutic heparin was used mainly in PICU. The duration of hospitalization was longer in MAS patients, especially those admitted to PICU. No children were treated with extracorporeal membrane oxygenation (ECMO) or renal replacement therapy. Two deaths were reported: one in a severely immunocompromised child and one in a previously healthy teenager with fulminant multiorgan dysfunction, with positive RT-PCR results for SARS-CoV-2. None of these patients fulfilled MAS criteria. Multivariate analysis of the influence of therapy on MAS, MAS-PICU, and the outcome showed that MAS alone is associated with incomplete recovery at discharge (Table 4; Supplementary Table 6).

**Table 4:** Multivariable analysis of the influence of therapy on the presence of macrophage activation syndrome (MAS), MAS requiring pediatric intensive care unit hospitalization (MAS-PICU), and outcome in 274 patients meeting MIS-C criteria.

**Parameter** **[analyzed value]**	**MAS *N* = 274**	**MAS-PICU** ***N* = 59**	**No-Complete recovery at discharge *N* = 235**
Age [1 y]	1.7 (1.1; 1.3); *P =* 0.0002	NA	NS
Glucocorticosteroids	3.2 (1.3; 7.7); *P =* 0.009	NA	NS
Heparin [therapeutic level]	NA	12.5 (2.3–68.0); *P =* 0.003	NS
Hospitalization time [>Median (10 d)]	2.3 (1.1; 4.9); *P =* 0.028	NS	NS
Mechanical ventilation	3.6 (0.93; 13.6); *P =* 0.063	NS	NS
MAS	NA	NA	4.0 (1.4–10.9); *P =* 0.008
Model summary (accuracy)	OR = 6.6; 79.9%; *P* < 0.0001 (96.3% non-MAS; 20.3% MAS)	OR = 11.2; 84.8% *P =* 0.002 (90% non-PICU; 55.6% PICU)	*P =* 0.010

## Discussion

Our study is one of the few published to date focusing on MAS in MIS-C patients. We found that 21% of MIS-C patients fulfilled 2016 Classification Criteria for Macrophage Activation Syndrome Complicating Systemic Juvenile Idiopathic Arthritis. Nevertheless, the outcome of MAS was less severe than we tend to observe in rheumatologic diseases, without requiring more immunomodulating treatment than recommended for MIS-C or PICU management. We have identified some indicators of MAS in MIS-C, such as symptoms characteristic of atypical KD, age within 5–12 years, skin erosion, and highly elevated inflammatory parameters and troponin levels. The overall clinical course and prognosis of MAS in MIS-C were better than expected based on the current knowledge on MAS complicating s-JIA, KD, or other rheumatological disorders. It suggests that these criteria might not apply to MIS-C.

There are several well-described phenotypes of MIS-C. Godfred-Cato et al. distinguished three non-exclusive categories of patients within MIS-C based on latent class analysis: Class 1 (named true” or classic MIS-C) with the highest degree of organ involvement and higher prevalence of shock and lymphopenia, with a little overlap with patients with KD; Class 2 (“acute COVID”) with the predominant respiratory symptoms and more common SARS-CoV-2 positivity by RT-PCR; and Class 3 (“KD-like”), with a phenotype similar to that of pre-pandemic KD (43–45). Rowley et al. confirmed different MIS-C phenotypes and underlined the need for basic science investigations into the host immune response to understand the pathogenesis of these (perhaps different) syndromes (44).

HLH and MAS are not discrete diseases but represent a continuum of hemophagocytic conditions that share a common pathway of impaired cytotoxicity leading to cytokine storm (46). Cytokine excess, particularly IL-18 and IFN-γ, is a common feature of HLH, MAS, and MIS-C. In our study, none of the children met the HLH-2004 criteria. HLH-2004 verification was biased by unavailable results of sCD25 level and NK cell count, which could influence our results. Another explanation of this finding could be immunopathological differences between these entities. A ratio of total IL-18/CXCL9 (C-X-C Motif Chemokine Ligand 9) has been used to differentiate patients with rheumatologic diseases and MAS from patients with HLH (47, 48). The levels of IL-18 reflect inflammasome activation, and CXCL9 indicates interferon-γ pathway activity. There is an impairment of granule-mediated cytotoxicity of natural killer (NK) cells in HLH. In MIS-C, the cytokine storm is linked mostly to INF-γ, which could explain a less severe disease course.

The amplitude of T cell activation and Th1 cytokines is higher in HLH versus MIS-C (48, 49). However, this comparison is of limited value, as it concerned primary HLH or EBV infection-associated HLH that fulfilled HLH-2004 diagnostic criteria, intended primarily for genetically conditioned HLH. MIS-C and HLH also differ in some clinical symptoms, such as cardiac dysfunction and gastrointestinal manifestation, which are typical for MIS-C but usually not in HLH. In addition, although low fibrinogen is a hallmark of HLH and MAS, we found a high level of fibrinogen (together with higher procalcitonin and ferritin) predictive of MAS criteria fulfillment. Considering all the above, we consider HLH-2004 criteria as suboptimal for MAS diagnosis in MIS-C.

Current data suggest distinct clinical features and laboratory differences between MIS-C and MAS (17, 50–54). The studies comparing MIS-C and MAS during s-JIA are restricted by small sample sizes (17, 52, 53). The cardiac, gastrointestinal, and neurological involvements and myalgia were more pronounced in MIS-C. In contrast to the poor prognosis of MAS in s-JIA, in our study, no deaths were reported in patients with MAS-MIS-C. Cytokine-targeting therapy is crucial in controlling hyperinflammation and is used widely in patients with MAS in children with s-JIA. Aggressive treatment is also suggested in patients with MIS-C and MAS, for example, a combination of IVIG plus pulses of methylprednisolone plus anakinra (55). None of the children in our group required such treatment. Children with MIS-C and MAS definitions' fulfillment were treated more often with intravenous immunoglobulins (94.8 vs. 87.6%) and steroids (87.3 vs. 62.7%). This finding is consistent with previous studies. Verdoni et al. diagnosed MAS according to 2016 Classification Criteria for Macrophage Activation Syndrome Complicating Systemic Juvenile Idiopathic Arthritis in five (50%) of ten patients with Kawasaki-like disease after the beginning of the SARS-CoV-2 epidemic. All patients were treated with IVIG and methylprednisolone with good clinical response. Two of them required inotropic agents (2). In our group, children diagnosed with MAS more often required mechanical ventilation (10.9 vs. 1.9%). The duration of hospitalization was longer in MIS-C and MAS patients, especially those admitted to PICU; 15% of children with MIS-C and MAS required PICU, while in other secondary hemophagocytic syndromes, the percentage of ICU patients reaches 38% (18, 30). Such difference indicates that MAS in MIS-C has a milder course. It may also be related to the more favorable course of MIS-C in the Polish population - a significantly lower percentage of hospitalizations in PICU was demonstrated (8%) than in reports from Western countries (60–80%), as shown in previous studies (5, 21). In addition, the nationwide MIS-C register and the high awareness of physicians, and thus early treatment, may also contribute to a better prognosis of patients with MAS during MIS-C. However, similar conclusions were reached by Otar et al., who found that patients with MIS-C had shorter hospitalization times than children with s-JIA-MAS. At the same time, they required PICU admission for myocarditis more frequently than children with s-JIA-MAS (53). There are also differences in basic laboratory tests. Patients with MIS-C had significantly lower lymphocyte count, higher CRP, erythrocyte sedimentation rate, and B-type natriuretic peptide levels. Moreover, ferritin concentration is elevated in MIS-C relative to healthy children, higher than in patients with KD but not as high as in patients with MAS complicating other diseases (17, 54).

The cytokine storm reflected in the laboratory findings of patients with MIS-C resembles MAS. However, differences in the degree of elevation of markers such as ferritin, IL-18, and CXCL9 suggest that the pathogenic cytokines in MIS-C differ from those driving MAS (17, 53). It is hypothesized that MIS-C resembles a more likely clinical state named pre-MAS (52). Based on cytokine profiles of children with MIS-C, Esteve-Sole A et al. proposed a more severe form of MIS-C (named MIS-C plus), closer to MAS both clinically and laboratory — related to higher IFN-γ levels (51). Five patients with MIS-C who had multiorgan involvement had higher levels of IFN-γ, IL-18, GM-CSF, RANTES, IP-10/CXCL10, IL-1α, and SDF-1. Authors conclude that such a cytokine profile might be an early sign of MAS.

On the other hand, MAS is not a specific diagnosis but a continuum of severity within MIS-C. Gurlevik et al. assessed the cytokine and chemokine profiles of children with MIS-C. They classified MIS-C patients into those with MAS and without MAS according to laboratory and clinical features of patients. They found that 13 (41.9%) of 31 MIS-C patients fulfilled 2016 Classification Criteria for Macrophage Activation Syndrome Complicating Systemic Juvenile Idiopathic Arthritis. Patients with MIS-C that met MAS criteria had higher levels of IL-17A and IFN-γ than those who did not fulfill the criteria; however, it was not statistically significant (40). Rodriguez-Smith et al. reported that eight (42%) of 19 patients with MIS-C who met MAS criteria (also based on 2016 MAS classification) had significantly higher CXCL9 concentration than patients with MIS-C without MAS. They conclude that the stratification of patients with MIS-C by high or low CXCL9 concentration supports MAS-like pathophysiology in patients with severe MIS-C. This finding might lead to a new approach to its diagnosis or management (41).

All these findings suggest that the MAS classification criteria used in MIS-C could correspond to a cytokine storm linked to IFN-γ (as in s-JIA). However, we think that they might still be non-specific. Based on further studies comparing MAS complicating s-JIA vs. MIS-C in the immunological and clinical context, the development and validation of the new definition and clinical criteria for identifying MAS in MIS-C might be required in further studies.

Our study has some limitations. The main one is the small patient population; however, to the best of our knowledge, this is the first study to evaluate and compare the clinical and laboratory characteristics of patients with MIS-C complicated by MAS in a larger cohort based on a multicenter registry. The minimal number of patients admitted to PICU limits the reliability of its risk factors assessment within patients with MAS in MIS-C. Moreover, we did not analyze the subgroup of patients with MIS-C (according to Godfred-Cato S et al. classification) but the whole group with different phenotypes. It would be interesting to classify the patients with MIS-C based on functional studies (INF-γ pathway) and specific cytokine profiles and to analyze these subgroups.

## Conclusions

The existing HLH and MAS diagnostic criteria do not address MIS-C. The 2016 Classification Criteria for Macrophage Activation Syndrome Complicating Systemic Juvenile Idiopathic Arthritis are easy to apply and help indicate children with a greater risk for more aggressive treatment requirements (including mechanical ventilation). In addition to the well-known signs of MAS (of which laboratory signs are the most prominent), we found that in patients with MIS-C, older age, atypical KD phenotype, and skin erosions are significant factors indicating risk for MAS. Highly elevated inflammatory parameters indicate MAS in children with MIS-C, and elevated troponin levels are predominantly a predictive factor for children with MAS requiring PICU hospitalization. The clinical course of MAS in MIS-C is milder, prognosis better, and treatment less aggressive than in MAS in the course of other diseases, including rheumatologic ones. However, MAS should be regarded not as a specific diagnosis but as a continuum of severity within MIS-C. Further studies are needed to define various aspects of MIS-C better and stratify it according to its severity. Further immunological profiling is required and could serve to choose optimal, individualized treatment.

## Data availability statement

The original contributions presented in the study are included in the article/Supplementary material, further inquiries can be directed to the corresponding author/s.

## Ethics statement

Ethical approval was obtained from the Bioethics Committee at the Wrocław Medical University (CWN UMW BW: 313/2020). Komisja Bioetyczna przy Uniwersytecie Medycznym we Wrocławiu 50-367 Wrocław, ul. J.Mikulicza-Radeckiego 4a tel.: 71 784 10 14, 71 784 17 10. Written informed consent to participate in this study was provided by the participants' legal guardian/next of kin.

## Author contributions

PB: conceptualization, methodology, validation, formal analysis, writing—original draft preparation, review, editing, visualization, supervision, and project administration. ES and DJ-L: methodology, validation, formal analysis, resources, software, data curation, writing—original draft preparation, review, editing, visualization, and supervision. EC: writing—original draft preparation. EG: methodology, formal analysis, and visualization. KL: methodology, resources, data curation, writing—original draft preparation, review, and editing, LS: resources, data curation, and writing—review and editing. MO-N and EK: resources, data curation, and methodology. JK: methodology, validation, formal analysis, visualization, and supervision. All authors contributed to the article and approved the submitted version.

## Conflict of interest

The authors declare that the research was conducted in the absence of any commercial or financial relationships that could be construed as a potential conflict of interest.

## Publisher's note

All claims expressed in this article are solely those of the authors and do not necessarily represent those of their affiliated organizations, or those of the publisher, the editors and the reviewers. Any product that may be evaluated in this article, or claim that may be made by its manufacturer, is not guaranteed or endorsed by the publisher.
