# Supplementary material for: Clinical characteristics of children with MIS-C fulfilling classification criteria for macrophage activation syndrome
Source: Front Pediatr. 2022 Sep 15;10:981711. doi: 10.3389/fped.2022.981711 (PMC9520614; doi:10.3389/fped.2022.981711)
Supplement: Supplementary file 1 [file Data_Sheet_1.docx]

**Table 1 supp**. Clinical characteristics of the MIS-C patients on admission, according to MAS and PICU.

| Characterictics | Group 1  (No-MAS)  N=215 | Group 2  (MAS)  N=59 | G2 vs G1  P value | G2 vs G1  P-value  age-adjusted | Group 3 (MAS-PICU patients)  N=50 | Group 4 (MAS-non-PICU patients)  N=9 | G4 vs G3  P value |
| --- | --- | --- | --- | --- | --- | --- | --- |
|  | n, n/N or Med  (% or IQR) | n, n/N or Med  (% or IQR) |  |  | n, n/N or Med  (% or IQR) | n, n/N or Med  (% or IQR) |  |
| Demographic parameters |  |  |  |  |  |  |  |
| Age, median | 8,1 (4,8;11,4) | 11,2 (8,3;13,5) | **<0,0001** | NA | 10,9 (8,3;13,5) | 12,6 (10,8;15,1) | 0,161 |
| Age groups |  |  |  |  |  |  |  |
| <5 y | 60/215 (27,9) | 4/59 (6,8) | **0,0002** |  | 4/50 (8,0) | 0/50 (0,0) | 0,538 |
| 5-12 y | 110/215 (51,2) | 30/59 (50,9) |  |  | 26/50 (52,0) | 4/9 (44,4) |  |
| 12-18y | 45/215 (20,9) | 25/59 (42,4) |  | NA | 20/50 (40,0) | 5/9 (55,6) |  |
| Sex | 130/215 (60,5) | 42/59 (71,2) | 0,131 | NA | 35/50 (70,0) | 7/9 (77,8) | 1,000 |
| Obesity [BMI>95th percentile for age and sex] | 13/184 (7,1) | 3/56 (5,4) | 0,654 | NA | 2/47 (4,3) | 1/9 (11,1) | 0,415 |
| Any comorbidity | 30/166 (18,1) | 8/43 (18,6) | 0,936 | NA | 7/36 (19,4) | 1/7 (14,3) | 1,000 |
| MAS criteria |  |  |  |  |  |  |  |
| Length of fever [days] | 6,0 (5,0; 8,0) | 6,0 (5,0; 8,0) | 0,241 | NA | 6,0 (5,0; 8,0) | 8,0 (6,0; 8,1) | 0,098 |
| Hepatomegaly | 3/215 (1,4) | 1/59 (1,7) | 0,865 | NA | 1/50 (2,0) | 0/9 (0,00) | 1,000 |
| Splenomegaly | 15/215 (7,0) | 6/59 (10,2) | 0,414 | NA | 5/50 (10,0) | 1/9 (11,1) | 1,000 |
| Kawasaki disease criteria fulfilled |  |  |  |  |  |  |  |
| typical | 19/215 (8,8) | 1/59 (1,7) | 0.087 | NA | 1/50 (2,0) | 0/9 (0,0) | 1,000 |
| atypical | 105/215 (48,8) | 45/59 (76,3) | 0,0002 | **0,00002** | 38 (76,0) | 7 (77,8) | 0,908 |
| typical or atypical | 124/215 (57,7) | 46/59 (78,0) | 0,004 | **0,0004** | 39 (78,0) | 7 (77,8) | 0,988 |
| Other signs and symptoms |  |  |  |  |  |  |  |
| Capillary refill time at admission >2s | 19/179 (10,6) | 11/45 (24,4) | 0,015 | 0,053 | 7/36 (19,4) | 4/9 (44,4) | 0,190 |
| Non-alert AVPU | 10/207 (4,8) | 7/55 (12,7) | 0.035 | **0,021** | 4/46 (8,7) | 3/9 (33,3) | 0,078 |
| Hypotension at admission | 19/167 (11,4) | 11/46 (23,9) | 0,030 | 0,111 | 6/38 (15,8) | 5/8 (62,5) | **0,013** |
| Rash | 168/207 (81,2) | 50/57 (87,7) | 0,248 | NA | 42/48 (87,5) | 8/9 (88,9) | 1,00 |
| Abdominal surgery performed | 7/100 (7,0) | 3/26 (11,5) | 0,446 | NA | 1/22 (4,6) | 2/4 (50,0) | 0,052 |
| Mucocutaneous and lymph nodes symptoms | 203/212 (95,8) | 59/59 (100,0) | 0,107 | NA | 50 /50 (100,00) | 9 /9(100,00) | NA |
| Gastrointestinal symptoms | 198/211 (93,8) | 52/59 (88,1) | 0,139 | NA | 44/50 (88,0) | 8/9 (88,9) | 1,000 |
| Osteoarticular and musuclar symptoms | 80/196 (40,8) | 31/55 (61,1) | 0,040 | 0,197 | 29/48 (60,4) | 2/7 (28,6) | 0,220 |
| Neurological symptoms | 172/205 (83,9) | 48/52 (92,3) | 0,123 | NA | 42/46 (91,3) | 6/6 (100,0) | 1,000 |
| Muscle hypotension | 19/208 (9,1) | 11/53 (20,8) | 0,018 | 0,063 | 11/46 (23,9) | 0/7 (0,00) | 0,322 |
| Upper repiratory symptoms | 71/202 (35,2) | 27/54 (50,0) | 0,042 | **0,034** | 23/46 (50,0) | 4/8 (50,0) | 1,000 |
| Lower respiratory symptoms | 95/200 (47,45) | 33/54 (61,1) | 0,076 | NA | 26/46 (56,5) | 7/8 (87,5) | 0,131 |
| Conjuctivitis | 165/210 (78,6) | 42 (76,4) | 0,724 | NA | 38/48 (79,2) | 4/7 (57,1) | 0,337 |
| Oral inflammation | 135/207 (65,2) | 38/55 (69,1) | 0,590 | NA | 32/47 (68,1) | 6/8 (75,0) | 1,000 |
| Hands and feet swelling or erythema | 102/205 (49,8) | 40/53 (75,5) | 0,0008 | **0,0002** | 34/45 (75,6) | 6/8 (75,0) | 1,000 |
| Cough | 59/206 (28,6) | 15/57 (26,3) | 0,730 | NA | 14/49 (28,6) | 1/8 (12,5) | 0,667 |
| Sore throat | 62/200 (31,0) | 24/53 (45,3) | 0,051 | NA | 21/45 (46, 7) | 3/8 (37,5) | 0,715 |
| Rhinities | 17/203 (8,4) | 6/53 (11,3) | 0,504 | NA | 5/45 (11,1) | 1/8 (12,5) | 1,000 |
| Breathing effort | 40/206 (19,4) | 23/55 (41,8) | 0,0006 | **0,002** | 17/47 (36,2) | 6/8 (75,0) | 0,057 |
| Swallowing difficulty | 28/199 (14,1) | 12/54 (22,2) | 0,145 | NA | 10/46 (21,7) | 2/8 (25,0) | 1,000 |
| Chest pain | 33/200 (16,5) | 15/53 (28,3) | 0,051 | NA | 13/46 (28,3) | 2/7 (28,6) | 1,000 |
| Arthritis | 7/204 (3,4) | 5/55 (9,1) | 0,076 | NA | 4/47 (8,5) | 1/8 (12,5) | 0,559 |
| Arthralgia | 32/203 (15,7) | 18/55 (32,7) | 0,005 | **0,015** | 17/48 (35,4) | 1/7 (14,3) | 0,406 |
| Muscle pain | 72/195 (36,9) | 31/56 (55,4) | 0,013 | 0,091 | 29/49 (59,2) | 2/7 (28,6) | 0,223 |
| Skin peeling on digits | 30/202 (14,9) | 14/52 (26,9) | 0,040 | **0,003** | 12/45 (26,7) | 2/7 (28,6) | 1,000 |
| Meningeal signs | 22/205 (10,7) | 5/55 (9,1) | 0,723 | NA | 4/47 (8,5) | 1/8 (12,5) | 0,559 |
| Lethargy | 115/200 (57,5) | 36/54 (66,7) | 0,185 | NA | 31/47 (66,0) | 5/7 (71,4) | 1,000 |
| Seizures | 2/203 (1,01) | 1/54 (1,9) | 0,598 | NA | 1/46 (2,2) | 0/8 (0,00) | 1,000 |
| Headache | 84/189 (44,4) | 28/54 (51,9) | 0,336 | NA | 25/46 (54,4) | 3/8 (37,5) | 0,460 |
| Peripheral nerve paralysis | 1/210 (0,48) | 1/55 (1,8) | 0,306 | NA | 1/47 (2,1) | 0/8 (0,00) | 1,000 |
| Nerve paresis | 1/204 (0,49) | 1/55 (1,8) | 0,380 | NA | 1/47 (2,1) | 0/8 (0,00) | 1,000 |
| Anosmia | 8/203 (4,02) | 0/55 (0,0) | 0,135 | NA | 0/47 (0,00) | 0/8 (0,00) | NA |
| Ageusia | 7/203 (3,5) | 0/54 (0,0) | 0,167 | NA | 0/47 (0,00) | 0/8 (0,00) | NA |
| Photophobia | 19/202 (9,4) | 9/53 (17,0) | 0,116 | NA | 9/47 (19,2) | 0/6 (0,00) | 0,574 |
| Annoyance | 85/198 (42,1) | 23/57 (40,4) | 0,815 | NA | 21/49 (42,9) | 2/8 (25,0) | 0,453 |
| Skin hyperesthesia | 69/201 (34,01) | 18/53 (34,0) | 0,960 | NA | 15/45 (33,3) | 3/8 (37,5) | 1,000 |
| Nausea | 127/204 (62,3) | 35/57 (61,4) | 0,907 | NA | 27/48 (56,3) | 8/9 (88,9) | 0,132 |
| Abdominal pain | 176/202 (87,1) | 46/58 (79,3) | 0,137 | NA | 38/49 (77,6) | 8/9 (88,9) | 0,668 |
| Diarrhoea | 126/208 (60,6) | 38/58 (65,5) | 0,494 | NA | 30/49 (61,2) | 8/9 (88,9) | 0,143 |
| Scrotum or labia swell | 17/205 (8,3) | 9/52 (17,3) | 0,054 | NA | 8/45 (17,8) | 1/7 (14,3) | 1,000 |
| Dysuria | 25/203 (12,3) | 15/54 (27,8) | 0,005 | **0,005** | 12/47 (25,5) | 3/7 (42,9) | 0,382 |
| Fluid refusal | 78/201 (38,8) | 18/55 (32,7) | 0,409 | NA | 17/48 (35,4) | 1/7 (14,3) | 0,406 |
| Skin erosions | 3/201 (1,5) | 5/52 (9,6) | 0,003 | **0,014** | 4/44 (9,1) | 1/8 (12,5) | 1,000 |
| Erythema at BCG vaccination site | 1/194 (0,53) | 0/53 (0,00) | 0,600 | NA | 0/46 (0,00) | 0/7 (0,00) | NA |
| Active bleeding | 4/206 (1,9) | 1/55 (1,9) | 1,000 | NA | 1/47 (2,1) | 0/8 (0,00) | 1,000 |
| Admission since symptoms start [d] | 5 (4; 6) | 5 (4; 6) | 0,930 | NA | 5 (4; 6) | 6 (4; 6) | 0,411 |
| Cervical lymphadenopathy | 70/199 (35,2) | 27/57 (47,4) | 0,094 | NA | 24/49 (49,0) | 3/8 (37,50) | 0,709 |
| Comorbidities |  |  |  |  |  |  |  |
| Any | 19/212 (9,0) | 6/55 (10,9) | 0,659 | NA | 6/47 (12,8) | 0/8 (0,00) | 0,577 |
| Rheumatic disease | 0/210 (0,0) | 1/54 (1,9) | 0,074 | NA | 1/46 (2,2) | 0/8 (0,00) | 1,000 |
| Hyptertension | 1/212 (0,48) | 0/55 (0,00) | 0,610 | NA | 0/47 (0,00) | 0/8 (0,00) | NA |
| CVD other than hypertension | 3/211 (1,4) | 1/55 (1,8) | 0,833 | NA | 1/47 (2,1) | 0/8 (0,00) | 1,000 |
| Asthma | 11/211 (5,2) | 0/55 (0,00) | 0,084 | NA | 0/47 (0,00) | 0/8 (0,00) | NA |
| Chronic lung disease | 1/211 (0,47) | 0/55 (0,00) | 0,609 | NA | 0/47 (0,00) | 0/8 (0,00) | NA |
| Diabetes | 1/212 (0,47) | 0/55 (0,00) | 0,610 | NA | 0/47 (0,00) | 0/8 (0,00) | NA |
| Oncological disease | 0/212 (0,00) | 1/55 (1,8) | 0,206 | NA | 1/47 (2,1) | 0/8 (0,00 | 1,000 |
| Hematological disease | 0/211 (0,00) | 1/55 (1,8) | 0,207 | NA | 1/47 (2,1) | 0/8 (0,00 | 1,000 |
| Immunosuppresion | 0/212 (0,00) | 1/54 (1,9) | 0,203 | NA | 1/46 (2,2) | 0/8 (0,00 | 1,000 |
| Chronic kidney disease | 1/212 (0,47) | 0/55 (0,00) | 1,000 | NA | 0/47 (0,00) | 0/8 (0,00 | NA |
| Chronic liver disease | 1/212 (0,47) | 0/55 (0,00) | 1,000 | NA | 0/47 (0,00) | 0/8 (0,00 | NA |
| Chronic neurological disease | 3/210 (1,4) | 3/54 (5,6) | 0,102 | NA | 3/46 (6,5) | 0/8 (0,00 | 1,000 |

CVD – cardiovascular disease

**Table 2 supp**. Laboratory results in the MIS-C patients at admission, according to MAS.

|  | no-MAS | | | | MAS | | | | Statistical analysis | |
| --- | --- | --- | --- | --- | --- | --- | --- | --- | --- | --- |
| Parameter | N | Med. | Q25 | Q75 | N | Med. | Q25 | Q75 | p-value | p-value  age adjusted |
| **C-reactive protein** concentration (CRP) [mg/L] | 214 | 129 | 80,43 | 176,1 | 57 | 189,08 | 106,9 | 265 | 0,002 | 0,028 |
| Erythrocyte sedimentation rate (ESR) [mm/h] | 96 | 46,5 | 32 | 68 | 29 | 44 | 29 | 65 | 0,387 | NA |
| Lactate [mmol/L] | 68 | 2 | 1,6 | 2,81 | 29 | 2,04 | 1,44 | 3,2 | 0,555 | NA |
| White blood cells (WBC) [10^3/µL] | 209 | 9,3 | 6,55 | 12,33 | 58 | 10,95 | 7 | 15,57 | 0,126 | NA |
| **Neutrophils [10^3/µL]** | 197 | 7,04 | 4,78 | 10 | 53 | 9,4 | 5,72 | 12,91 | 0,015 | 0,020 |
| **Lymphocytes [10^3/µL]** | 203 | 1,16 | 0,7 | 2,02 | 54 | 0,74 | 0,48 | 1,1 | <0,0001 | 0,017 |
| Hematocrit (hct) [%] | 211 | 33,8 | 30,6 | 36,4 | 57 | 34,2 | 31,1 | 37 | 0,388 | NA |
| Hemoglobin (hgb) [g/dL] | 210 | 11,6 | 10,6 | 12,6 | 57 | 12,2 | 11 | 13,3 | 0,056 | NA |
| **Platelets (PLT) [10^3/µL]** | 209 | 188 | 140 | 268 | 58 | 140 | 95 | 189 | <0,0001 | 0,001 |
| **Procalcitonin [ng/mL]** | 198 | 1,855 | 0,89 | 4,94 | 52 | 8,35 | 2,48 | 22,39 | <0,0001 | 0,0001 |
| **Ferritin [µg/L]** | 172 | 292,3 | 169,65 | 411,5 | 51 | 920,13 | 726,1 | 1309 | <0,0001 | <0,0001 |
| **D-dimers [mg/L]** | 193 | 2,4 | 1,322 | 4,3 | 54 | 3,78 | 2,13 | 6,98 | 0,0001 | 0,012 |
| INR [1] | 182 | 1,22 | 1,1 | 1,35 | 54 | 1,24 | 1,14 | 1,42 | 0,455 | NA |
| Activated partial thromboplastin time (APTT) [s] | 175 | 36 | 32 | 39,2 | 51 | 33,8 | 29,6 | 39,6 | 0,405 | NA |
| Aspartate transaminaze (AspAT) [U/L] | 198 | 32 | 24 | 48 | 58 | 38,5 | 30 | 78 | 0,006 | 0,276 |
| Alanine transaminaze (AlAT) [U/L] | 206 | 23 | 15 | 35 | 58 | 36 | 20 | 74 | 0,0002 | 0,115 |
| Plasma bilirubin [mg/dL] | 109 | 0,5 | 0,33 | 0,77 | 44 | 0,75 | 0,46 | 1,45 | 0,004 | 0,582 |
| **Sodium** [mmol/L] | 205 | 135 | 133 | 137 | 55 | 132 | 131 | 137 | 0,002 | 0,005 |
| Glucose [mg/dL] | 185 | 99,8 | 87 | 115 | 47 | 107 | 91 | 126 | 0,134 | NA |
| **Brain** natriuretic peptide (BNP) [pg/mL] | 37 | 275 | 114 | 659,7 | 15 | 1933,8 | 210,7 | 4392,3 | 0,006 | 0,052 |
| N-terminal pro-BNP (NT-proBNP) [pg/mL] | 128 | 1992,5 | 607,3 | 5943,5 | 31 | 5953 | 1256 | 20747 | 0,023 | 0,053 |
| Fibrinogen [g/L] | 167 | 5,42 | 4,5 | 6,38 | 53 | 5,72 | 4,18 | 7 | 0,615 | NA |
| Lactate dehydrogenase (LDH) [U/L] | 165 | 272 | 227 | 323 | 54 | 286 | 231 | 412 | 0,183 | NA |
| Triglicerides [mg/dL] | 113 | 140 | 115 | 190 | 44 | 210 | 133,2 | 293,5 | 0,001 | 0,000 |
| Amylase [U/L] | 91 | 26 | 19 | 38 | 39 | 28 | 20 | 38 | 0,899 | NA |
| **Serum** creatinine [mg/dL] | 198 | 0,48 | 0,35 | 0,64 | 58 | 0,65 | 0,48 | 1,02 | <0,0001 | 0,033 |
| Creatine kinase (CK) [U/L] | 53 | 52 | 38 | 92 | 16 | 43 | 32,45 | 81 | 0,455 | NA |
| **Urea [mg/dL]** | 187 | 22 | 17 | 29 | 57 | 27 | 21,4 | 57,1 | 0,001 | 0,005 |
| **Gamma glutamyl transpeptidase (GGTP) [U/L]** | 100 | 20 | 13 | 43,5 | 40 | 40 | 19 | 99 | 0,002 | 0,049 |
| IL-6 [pg/mL] | 56 | 106,5 | 26,3 | 385,5 | 12 | 97,3 | 47,7 | 235,3 | 0,994 | NA |
| **Albumins** [g/dL] | 187 | 3,4 | 2,88 | 3,785 | 52 | 3,1 | 2,5 | 3,65 | 0,011 | 0,003 |
| Total protein [g/dL] | 129 | 5,95 | 5,2 | 6,5 | 36 | 5,6 | 4,85 | 6,2 | 0,056 | NA |
| Sterile leucocyturia | 202 | 0 | 0 | 0 | 58 | 0 | 0 | 1 | 0,111 | NA |
| Aseptic meningitis | 78 | 0 | 0 | 0 | 18 | 0 | 0 | 0 | 0,616 | NA |
| Elevated level of troponin * | 170 | 38 (22,0) | | | 52 | 24 (46,0) | | | 0,008 | 0,051 |
| eGFR | 172 | 113,3 | 92,9 | 134,8 | 55 | 104,6 | 63,16 | 125,6 | 0,005 | 0,362 |

*n (%); NA-not analyzed

**Table 3**.suppl Laboratory results in the MIS-C patients at admission, according to PICU.

| Parameter | MAS no-PICU | | | | MAS PICU | | | | p-value |
| --- | --- | --- | --- | --- | --- | --- | --- | --- | --- |
|  | N | Med. | Q25 | Q75 | N | Med. | Q25 | Q75 |  |
| C-reactive protein concentration (CRP) [mg/L] | 48 | 167,73 | 102,35 | 241,1 | 9 | 282 | 206 | 295 | 0,088 |
| Erythrocyte sedimentation rate (ESR) [mm/h] | 27 | 43 | 28 | 61 | 2 | 81 | 77 | 85 | 0,058 |
| Lactate [mmol/L] | 21 | 2,04 | 1,44 | 3,2 | 8 | 2,1 | 1,45 | 4,25 | 0,714 |
| **White blood cells (WBC) [10^3/µl]** | 49 | 10,24 | 6,38 | 14,41 | 9 | 13,51 | 11,58 | 21 | 0,032 |
| **Neutrophils [10^3/µl]** | 44 | 8,56 | 4,97 | 12,86 | 9 | 12,4 | 10,19 | 20 | 0,021 |
| Lymphocytes [10^3/µl] | 46 | 0,76 | 0,47 | 1,18 | 8 | 0,73 | 0,61 | 0,87 | 0,827 |
| Hematocrit (hct) [%] | 48 | 34,1 | 31,2 | 36,65 | 9 | 34,9 | 31,1 | 39 | 0,948 |
| Hemoglobin (hgb) [g/dL] | 48 | 12,2 | 10,95 | 13,25 | 9 | 12 | 11,2 | 13,3 | 0,939 |
| Platelets (PLT) [10^3/µl] | 49 | 132 | 95 | 193 | 9 | 151 | 134 | 186 | 0,541 |
| **Procalcitonin [ng/ml]** | 43 | 6,1 | 1,28 | 16 | 9 | 49,87 | 15,96 | 58 | 0,004 |
| Ferritin [µg/L] | 42 | 862 | 739 | 1182 | 9 | 944,5 | 687 | 1335 | 0,776 |
| D-dimers [mg/L] | 45 | 3,7 | 1,99 | 6,98 | 9 | 5,13 | 3,71 | 6,27 | 0,444 |
| INR [1] | 45 | 1,25 | 1,14 | 1,42 | 9 | 1,23 | 1,15 | 1,31 | 1,000 |
| Activated partial thromboplastin time (APTT) [s] | 42 | 33,7 | 29,6 | 38 | 9 | 41 | 31,5 | 41,91 | 0,129 |
| Aspartate transaminaze (AspAT) [U/L] | 49 | 43 | 30 | 78 | 9 | 32 | 25 | 48 | 0,459 |
| Alanine transaminaze (AlAT) [U/L] | 49 | 37 | 21 | 74 | 9 | 32 | 16 | 60 | 0,805 |
| Plasma bilirubin [mg/dL] | 37 | 0,64 | 0,45 | 1,49 | 7 | 0,8 | 0,7 | 1,1 | 0,642 |
| Sodium [mmol/L] | 46 | 132 | 131 | 136 | 9 | 133 | 130 | 139 | 0,691 |
| Glucose [mg/dL] | 39 | 109 | 97 | 130 | 8 | 90,5 | 84 | 109 | 0,054 |
| Brain natriuretic peptide (BNP) [pg/mL] | 11 | 798 | 139,2 | 9957,5 | 4 | 2017,4 | 1657,4 | 3246,65 | 0,648 |
| **N-terminal pro-BNP (NT-proBNP) [pg/mL]** | 26 | 3174 | 1069 | 13926 | 5 | 32672 | 9347 | 40650 | 0,019 |
| **Fibrinogen [g/L]** | 44 | 5,51 | 4,07 | 6,4 | 9 | 7,14 | 6,26 | 7,83 | 0,022 |
| Lactate dehydrogenase (LDH) [U/L] | 46 | 286 | 208 | 400 | 8 | 278 | 248 | 508,5 | 0,715 |
| Triglicerides [mg/dL] | 38 | 202,5 | 131 | 277 | 6 | 249 | 172 | 310 | 0,527 |
| Amylase [U/L] | 33 | 24 | 20 | 32 | 6 | 35,5 | 31 | 49 | 0,073 |
| **Serum creatinine [mg/dL**] | 49 | 0,57 | 0,46 | 0,82 | 9 | 1,4 | 0,9 | 2,7 | 0,001F |
| Creatine kinase (CK) [U/L] | 14 | 45,5 | 38 | 104 | 2 | 14,95 | 2 | 27,9 | 0,068 |
| **Urea [mg/dL]** | 48 | 25,5 | 18 | 55 | 9 | 61 | 34 | 102 | 0,004F |
| Gamma glutamyl transpeptidase (GGTP) [U/L] | 33 | 40 | 20 | 114 | 7 | 30 | 16 | 90 | 0,423 |
| IL-6 [pg/mL] | 11 | 87,62 | 36,49 | 289 | 1 | 143 | 143 | 143 | 1,000 |
| Albumins [g/dL] | 43 | 3,2 | 2,58 | 3,7 | 9 | 2,7 | 2,31 | 3 | 0,113 |
| Total protein [g/dL] | 28 | 5,67 | 4,85 | 6,2 | 8 | 5,3 | 4,85 | 6,25 | 0,834 |
| Sterile leucocyturia | 49 | 0 | 0 | 1 | 9 | 0 | 0 | 0 | 0,772 |
| Aseptic meningitis | 14 | 0 | 0 | 0 | 4 | 0 | 0 | 0 | 0,958 |
| **Elevated level of troponin*** | 43 | 15 (35) | | | 9 | 9 (100) | | | 0,0004F^A^ |
| **eGFR** | 46 | 108,5 | 75,4 | 129,1 | 9 | 51,6 | 27,99 | 75,81 | 0,001F |

*n (%); A-OR=31,2 (1,7-578,7);

**Table 4 suppl.** Laboratory results (at respective peak) in the MIS-C patients, according to MAS.

| Parameter | no-MAS  N=215 | | | | MAS  N=59 | | | | Statistical analysia | |
| --- | --- | --- | --- | --- | --- | --- | --- | --- | --- | --- |
|  | N | Med. | Q25 | Q75 | N | Med. | Q25 | Q75 | p-value | p-value (age-adjusted) |
| CRP at its maximum [mg/L] | 209 | 155,6 | 90,1 | 229,7 | 55 | 210,9 | 120,9 | 306,0 | 0,013 | 0,107 |
| ESR at its maximum [mm] | 101 | 60,0 | 38,0 | 81,0 | 35 | 48,0 | 29,0 | 77,0 | 0,230 | NA |
| Serum lactates at its maximum [mmol/L] | 82 | 2,4 | 1,7 | 3,4 | 29 | 3,1 | 2,0 | 4,0 | 0,121 | NA |
| **WBC** at its maximum [10^3/µl] | 203 | 13,6 | 10,3 | 18,0 | 57 | 16,5 | 12,1 | 23,9 | 0,001 | 0,001 |
| WBC at its minimum [10^3/µl] | 187 | 6,7 | 5,2 | 9,0 | 51 | 6,2 | 4,5 | 9,2 | 0,754 | NA |
| Neutrophils at its maximum [10^3/µl] | 198 | 9,4 | 6,7 | 13,0 | 56 | 12,8 | 9,1 | 19,5 | 0,0001 | 0,001 |
| Lymphocytes at its minimum [10^3/µl] | 202 | 1,2 | 0,7 | 2,0 | 55 | 0,6 | 0,4 | 0,9 | <0,0001 | 0,001 |
| Hct at its minimum [%] | 210 | 29,9 | 27,3 | 32,5 | 57 | 29,4 | 26,4 | 31,0 | 0,121 | NA |
| Hbg at its minimum [g/dL] | 210 | 10,3 | 9,5 | 11,2 | 57 | 10,2 | 9,2 | 10,9 | 0,189 | NA |
| PLT at its maximum [10^3/µl] | 209 | 547,0 | 386,0 | 750,0 | 56 | 503,5 | 369,5 | 607,5 | 0,061 | NA |
| PLT at its minimum [10^3/µl] | 207 | 176,0 | 120,0 | 255,0 | 57 | 105,0 | 78,0 | 152,0 | <0,0001 | 0,0001 |
| Procalcitonin at its maximum [ng/ml] | 197 | 3,1 | 1,2 | 8,0 | 57 | 13,0 | 4,6 | 22,0 | <0,0001 | 0,008 |
| Ferritin at its maximum [µg/L] | 184 | 317,8 | 184,5 | 444,0 | 56 | 1109,1 | 807,5 | 1423,5 | <0,0001 | <0,0001 |
| D-dimers at its maximum [mg/L] | 197 | 3,4 | 1,7 | 5,7 | 56 | 4,8 | 2,7 | 10,7 | 0,000 | 0,027 |
| INR at its maximum | 187 | 1,2 | 1,1 | 1,4 | 55 | 1,3 | 1,2 | 1,5 | 0,021 | 0,790 |
| APTT at its maximum [s] | 188 | 36,2 | 31,8 | 40,6 | 53 | 37,2 | 30,4 | 42,4 | 0,598 | NA |
| Antitrombin at its maximum [%] | 31 | 83,0 | 71,0 | 95,0 | 11 | 64,0 | 56,0 | 92,0 | 0,141 | NA |
| ASPAT at its maximum [U/L] | 199 | 40,0 | 29,0 | 57,1 | 57 | 63,0 | 44,0 | 91,0 | <0,0001 | 0,001 |
| AlAT at its maximum [U/L] | 201 | 29,0 | 19,0 | 52,0 | 57 | 55,0 | 37,0 | 90,0 | <0,0001 | 0,043 |
| Bilirubin at its maximum [mg/dL] | 124 | 0,5 | 0,4 | 0,8 | 46 | 0,9 | 0,5 | 1,4 | 0,001 | 0,154 |
| Serum sodium at its minimum [mmol/L] | 204 | 134,0 | 132,0 | 136,0 | 55 | 132,0 | 129,0 | 133,0 | <0,0001 | <0,0001 |
| Serum glucose at its maximum [mg/dL] | 185 | 107,0 | 95,0 | 134,0 | 49 | 130,0 | 112,7 | 153,0 | <0,0001 | 0,002 |
| Serum glucose at its minimum [mg/dL] | 169 | 87,0 | 77,0 | 97,0 | 46 | 85,0 | 77,0 | 105,0 | 0,723 | NA |
| BNP at its maximum [pg/mL] | 105 | 1274,0 | 315,0 | 5031,0 | 36 | 8788,5 | 1779,0 | 21234,0 | 0,000 | 0,0004 |
| NT-ProBNP at its maximum [pg/mL] | 72 | 4616,0 | 2029,5 | 11721,0 | 17 | 6340,0 | 5731,0 | 7768,0 | 0,140 | NA |
| Fibrinogen at its maximum [g/L] | 172 | 5,6 | 4,6 | 6,7 | 56 | 5,7 | 4,4 | 7,0 | 0,819 | NA |
| Fibrinogen at its minimum [g/L] | 166 | 3,3 | 2,4 | 4,3 | 54 | 2,3 | 1,8 | 2,8 | <0,0001 | 0,0001 |
| LDH at its maximum [U/L] | 168 | 289,0 | 239,0 | 328,5 | 54 | 312,0 | 239,0 | 440,0 | 0,049 | 0,062 |
| Triglicerides at its maximum [mg/dL] | 127 | 153,1 | 124,0 | 221,0 | 49 | 207,0 | 136,0 | 318,0 | 0,006 | 0,0008 |
| Amylase at its maximum [U/L] | 101 | 37,0 | 22,0 | 53,0 | 38 | 32,5 | 25,0 | 62,0 | 0,648 | NA |
| Serum creatinine at its maximum [mg/dL] | 197 | 0,5 | 0,4 | 0,6 | 56 | 0,7 | 0,5 | 1,2 | <0,0001 | 0,002 |
| CK at its maximum [U/L] | 58 | 56,5 | 37,0 | 92,0 | 17 | 45,0 | 37,0 | 61,0 | 0,433 | NA |
| Urea at its maximum [mg/dL] | 187 | 27,0 | 20,0 | 35,0 | 57 | 38,4 | 29,0 | 68,0 | <0,0001 | <0,0001 |
| GGTP at its maximum [U/L] | 114 | 22,5 | 14,0 | 69,0 | 46 | 60,0 | 29,0 | 122,0 | <0,0001 | 0,010 |
| IL-6 at its maximum [pg/mL] | 32 | 148,5 | 54,3 | 615,4 | 13 | 198,3 | 36,5 | 695,9 | 0,990 | NA |
| Albumins at its maximum [g/dL] | 179 | 2,9 | 2,6 | 3,4 | 49 | 2,5 | 2,3 | 3,0 | 0,0002 | 0,001 |
| Total protein at its maximum [g/dL] | 125 | 5,7 | 5,1 | 6,5 | 31 | 5,4 | 4,5 | 6,2 | 0,070 | NA |
| Urine protein at its maximum [mg/dL]* | 106 | 64 (60,4) | | | 33 | 25 (75,8) | | | 0,107 | NA |
| Sterile leucocyturia at respective peak* | 196 | 24 (12,2) | | | 55 | 17 (30,9) | | | 0,009 | 0,009 |
| Aseptic Meningitis at respective peak* | 89 | 6 (6,7) | | | 18 | 0 (0,0) | | | 0,587 | NA |
| Elevated level of troponin at its max* | 127 | 60 (47,2) | | | 52 | 32 (61,5) | | | 0,082 | NA |
| eGFR at its minimum | 174 | 107,5 | 86,7 | 128,5 | 53 | 85,6 | 53,4 | 118,4 | 0,001 | 0,016 |

*n (%)

**Table 5 suppl.** Laboratory results (at respective peak) in the MIS-C patients, according to PICU.

| Parameter | MAS no-PICU, N=50 | | | | MAS PICU; N=9 | | | | p-value |
| --- | --- | --- | --- | --- | --- | --- | --- | --- | --- |
|  | N | Med. | Q25 | Q75 | N | Med. | Q25 | Q75 |  |
| CRP at its maximum [mg/L] | 47 | 196 | 120,9 | 278,31 | 8 | 288,1 | 143,65 | 355,97 | 0,247 |
| ESR at its maximum [mm] | 31 | 45 | 29 | 70 | 4 | 81 | 49,5 | 90 | 0,233 |
| Serum lactates at its maximum [mmol/L] | 22 | 3 | 1,9 | 3,6 | 7 | 3,5 | 2 | 5 | 0,161 |
| WBC at its maximum [10^3/µl] | 48 | 15,55 | 11,77 | 22,25 | 9 | 28,42 | 19,78 | 29 | 0,019 |
| WBC at its minimum [10^3/µl] | 45 | 6,22 | 4,52 | 8,83 | 6 | 7,28 | 5,5 | 9,15 | 0,682 |
| Neutrophils at its maximum [10^3/µl] | 47 | 12,48 | 8,81 | 17,5 | 9 | 22 | 12,4 | 25,12 | 0,032 |
| Lymphocytes at its minimum [10^3/µl] | 46 | 0,6 | 0,39 | 0,96 | 9 | 0,6 | 0,43 | 0,7 | 0,964 |
| Hct at its minimum [%] | 48 | 29,4 | 26,95 | 31 | 9 | 28,9 | 23 | 30,5 | 0,289 |
| Hgb at its minimum [g/dL] | 48 | 10,2 | 9,2 | 11,05 | 9 | 9,8 | 8,8 | 10,7 | 0,584 |
| PLT at its maximum [10^3/µl] | 47 | 509 | 378 | 646 | 9 | 479 | 305 | 512 | 0,384 |
| PLT at its minimum [10^3/µl] | 48 | 104,5 | 78,5 | 147,5 | 9 | 141 | 78 | 184 | 0,370 |
| Procalcitonin at its maximum [ng/ml] | 48 | 11,49 | 3,88 | 17,92 | 9 | 49,87 | 18,38 | 58,15 | 0,003 |
| Ferritin at its maximum [µg/L] | 47 | 1101 | 804 | 1437 | 9 | 1128,5 | 944,5 | 1335 | 0,704 |
| D-dimers at its maximum [mg/L] | 47 | 4,58 | 2,67 | 10,81 | 9 | 5,77 | 3,77 | 9 | 0,475 |
| INR at its maximum | 46 | 1,3 | 1,19 | 1,5 | 9 | 1,4 | 1,23 | 1,51 | 0,426 |
| APTT at its maximum [s] | 44 | 34,75 | 29,8 | 40,45 | 9 | 41,9 | 40 | 44,54 | 0,012 |
| Antitrombin at its maximum [%] | 7 | 64 | 59 | 92 | 4 | 64,16 | 54,7 | 87,46 | 0,777 |
| ASPAT at its maximum [U/L] | 48 | 64 | 44,05 | 88,5 | 9 | 55 | 44 | 117 | 0,686 |
| AlAT at its maximum [U/L] | 48 | 54,3 | 36,5 | 88,5 | 9 | 61 | 38 | 91 | 0,861 |
| Bilirubin at its maximum [mg/dL] | 39 | 0,66 | 0,49 | 1,49 | 7 | 0,9 | 0,7 | 1,1 | 0,418 |
| Serum sodium at its minimum [mmol/L] | 46 | 132 | 129 | 133 | 9 | 131,1 | 130 | 134 | 0,909 |
| Serum glucose at its maximum [mg/dL] | 41 | 130 | 112 | 150 | 8 | 135,4 | 118,5 | 228 | 0,417 |
| Serum glucose at its minimum [mg/dL] | 39 | 86 | 79 | 107 | 7 | 76 | 65 | 95 | 0,087 |
| BNP at its maximum [pg/mL] | 29 | 9821 | 969 | 15472 | 7 | 4392,3 | 1933,8 | 32672 | 0,968 |
| NT-ProBNP at its maximum [pg/mL] | 15 | 6340 | 3006 | 7768 | 2 | 31730,5 | 5953 | 57508 | 0,412 |
| Fibrinogen at its maximum [g/L] | 48 | 5,64 | 4,43 | 6,95 | 8 | 6,4 | 4,52 | 7,49 | 0,656 |
| Fibrinogen at its minimum [g/L] | 46 | 2,17 | 1,8 | 2,8 | 8 | 2,47 | 2,14 | 3,43 | 0,361 |
| LDH at its maximum [U/L] | 46 | 312 | 231 | 412 | 8 | 329 | 257,5 | 508,5 | 0,488 |
| Triglicerides at its maximum [mg/dL] | 42 | 202 | 136 | 314 | 7 | 237 | 135 | 519 | 0,689 |
| Amylase at its maximum [U/L] | 32 | 32 | 24 | 58,5 | 6 | 46,5 | 39 | 111 | 0,075 |
| Serum creatinine at its maximum [mg/dL] | 47 | 0,6 | 0,48 | 1,02 | 9 | 1,4 | 0,9 | 2,7 | 0,003 |
| CK at its maximum [U/L] | 15 | 46 | 37 | 104 | 2 | 17,45 | 7 | 27,9 | 0,062 |
| Urea at its maximum [mg/dL] | 48 | 34,5 | 26 | 63,5 | 9 | 85 | 68 | 126 | 0,001 |
| GGTP at its maximum [U/L] | 38 | 55 | 29 | 123 | 8 | 60,15 | 29 | 103,5 | 0,739 |
| IL-6 at its maximum [pg/mL] | 13 | 198,3 | 36,49 | 695,9 | 0 | 0 | 0 | 0 | NA |
| Albumins at its maximum [g/dL] | 42 | 2,53 | 2,3 | 3 | 7 | 2,5 | 2,3 | 2,7 | 0,742 |
| Total protein at its maximum [g/dL] | 26 | 5,45 | 4,6 | 6,16 | 5 | 4,3 | 4,2 | 4,9 | 0,107 |
| Urine protein at its maximum [mg/dL]* | 27 | 21 (77,8) | | | 6 | 4 (66,7) | | | 0,566 |
| Sterile leucocyturia at respective peak* | 46 | 15 (32,6) | | | 9 | 2 (22,2) | | | 0,537 |
| Aseptic Meningitis at respective peak* | 14 | 0 (0,0) | | | 4 | 0 (0,0) | | | NA |
| Elevated level of troponin at its max* | 43 | 23 (53,5) | | | 9 | 9 (100,0) | | | 0,009 |
| eGFR at its minimum | 44 | 103,51 | 63,92 | 125,18 | 9 | 51,55 | 27,99 | 70,21 | 0,003 |

*n (%)

**Table 6 suppl**. Therapy and outcome in the MIS-C patients, according to MAS and PICU

| Characterictics  n/N (%) or Med (Q1 ;Q3) | Group 1  (No-MAS)  N=215 | Group 2  (MAS)  N=59 | P value  (Chi2 or Fisher exact test or U Mann-Whitney test) | P-value  age-adjusted | Group 3  (MAS-non-PICU)  N=50 | Group 4  (MAS- PICU)  N=9 | P value  (Chi2 or Fisher exact test or U Mann-Whitney test) |
| --- | --- | --- | --- | --- | --- | --- | --- |
| IVIG | 185/211 (7,7) | 55/58 (94,8) | 0,120 | NA | 47/49 (95,9) | 8/9 (88,9) | 0,381 |
| GCS | 126/201 (62,7) | 48/55 (87,3) | 0,0005F | **0,003** | 40/46 (87,0) | 8/9 (88,9) | 0,874 |
| ASA | 177/207 (85,5) | 49/58 (84,5) | 0,846 | NA | 41/49 (83,7) | 8/9 (88,9) | 1,00F |
| Heparin (therapeutic level) | 13/185 (7,0%) | 10/53 (19,0) | 0,010F | 0,082 | 5/45 (11,1) | 5/8 (62,5) | **0,004F** |
| IL-1 inhibitors (anakinra) | 0/215 (0,00) | 0/59 (0,00) | NA | NA | 0/50 (0,00) | 0/9 (0,00) | NA |
| IL-6 inhibitors (tocilizumab) | 1/176 (0,57) | 0/40 (0,00) | 0,633 | NA | 0/40 (0,00) | No data | NA |
| TNF-alpha inhibitors (infliximab) | 0/215 (0,00) | 0/59 (0,00) | NA | NA | 0/50 (0,00) | 0/9 (0,00) | NA |
| Mechanical ventilation | 4/206 (1,9) | 6/55 (10,9) | 0,008 | **0,019** | 0/46 (0,00) | 6/9 (66,7) | **0,0001F** |
| Inotropic agents | no data | no data | no data | NA | no data | no data | NA |
| Hospitalization time | 12 (10; 13)/215 | 20 (14; 25)/59 | 0,0003 | **0,004** | 10 (8; 12)/59 | 12 (10; 14)/9 | **0,021** |
| PICU | 14/215 (6,5) | 9/59 (15,3) | 0,036F | 0,198 | 0/50 (0,00) | 9/9 (100%) | NA |
| Complete recovery at discharge | 178/187 (95,2) | 40/48 (83,33) | 0,010 | **0,013** | 33/40 (82,5) | 7/8 (87,5) | 1,000 |
| Legend: GCS – glucocorticosteroids, IQR: interquartile range, IVIG – intravenous immunoglobulins, Med: median, PICU – pediatric intensive care unit. *p values < 0.05.; F-Fisher exact test | | | | | | | |
